# Population receptive field models capture the event-related magnetoencephalography response with millisecond resolution

**DOI:** 10.1162/imag_a_00285

**Published:** 2024-09-18

**Authors:** Katharina Eickhoff, Arjan Hillebrand, Maartje C. de Jong, Serge O. Dumoulin

**Affiliations:** Spinoza Centre for Neuroimaging, Amsterdam, The Netherlands; Department of Computational Cognitive Neuroscience and Neuroimaging, Netherlands Institute for Neuroscience, Amsterdam, The Netherlands; Department of Experimental and Applied Psychology, Vrije Universiteit Amsterdam, Amsterdam, The Netherlands; Department of Clinical Neurophysiology and Magnetoencephalography Centre, Amsterdam UMC, Amsterdam, The Netherlands; Amsterdam Neuroscience, Brain Imaging, Amsterdam, The Netherlands; Amsterdam Neuroscience, Systems and Network Neuroscience, Amsterdam, The Netherlands; Department of Psychology, University of Amsterdam, Amsterdam, The Netherlands; Department of Experimental Psychology, Utrecht University, Utrecht, The Netherlands

**Keywords:** spatiotemporal resolution, population receptive fields, visual cortex, ultra-high-field fMRI, MEG

## Abstract

Much of the visual system is organized into visual field maps. In humans, this organization can be studied non-invasively by estimating the receptive fields of populations of neurons (population receptive fields; pRFs) with functional magnetic resonance imaging (fMRI). However, fMRI cannot capture the temporal dynamics of visual processing that operate on a millisecond scale. Magnetoencephalography (MEG) does provide this temporal resolution but generally lacks the required spatial resolution. Here, we introduce a forward modeling approach that combines fMRI and MEG, enabling us to estimate pRFs with millisecond resolution. Using fMRI, we estimated the participant’s pRFs using conventional pRF-modeling. We then combined the pRF models with a forward model that transforms the cortical responses to the MEG sensors. This enabled us to predict event-related field responses measured with MEG while the participants viewed brief (100 ms) contrast-defined bar and circle shapes. We computed the goodness of fit between the predicted and measured MEG responses across time using cross-validated variance explained. We found that the fMRI-estimated pRFs explained up to 91% of the variance in individual MEG sensor’s responses. The variance explained varied over time and peaked between 75 ms to 250 ms after stimulus onset. Perturbing the pRF positions decreased the explained variance, suggesting that the pRFs were driving the MEG responses. In conclusion, pRF models can predict event-related MEG responses, enabling routine investigation of the spatiotemporal dynamics of human pRFs with millisecond resolution.

## 1 Introduction

How the visual system integrates visual information that reaches our retina is quite remarkable. Each individual neuron in the visual cortex responds to only a confined part of the visual field, its so-called receptive field (Hubel & Wiesel, 1959). Through communication and recurrent processing across neurons along the visual hierarchy, a coherent image is formed and we can see the world around us in a sensible manner (Angelucci & Bressloff, 2006;V. A. F. Lamme & Roelfsema, 2000). Information processing happens exceptionally fast. Within tens of milliseconds, visual information reaches the primary visual cortex and other visual areas (Bullier, 2001). Information is then propagated along the hierarchy of visual regions and relayed through horizontal and feedback connections within and across regions (V. Lamme, 1995).

Much of the visual system is organized into visual field maps (Wandell et al., 2007). The visual field map properties of individual neurons can be studied invasively using electrophysiological tools (Hubel & Wiesel, 1959). To measure receptive field in the human brain, however, we generally rely on non-invasive methods, such as functional resonance imaging (fMRI), which measures populations of neurons inside a voxel (i.e., 3D pixel containing the signal from a patch of cortex). We can measure the aggregate receptive field properties of these populations of neurons using population receptive field (pRF) modeling (Dumoulin & Knapen, 2018;Dumoulin & Wandell, 2008;Wandell & Winawer, 2015), which estimates the region of visual space that a voxel responds to.

The visual system is fast and dynamic, however, and standard fMRI protocols are slow in comparison, in the range of seconds. This is due to the slow nature of the hemodynamic response that constitutes the blood-oxygen-level-depended (BOLD) response measured with fMRI (Logothetis & Wandell, 2004). Other non-invasive techniques, such as magnetoencephalography (MEG), provide temporal resolution in the order of milliseconds, but generally lack the spatial resolution to study the detailed layout of the pRFs (Hämäläinen et al., 1993). Previous studies have tried to solve the lack of spatial resolution by means of source reconstruction (Brookes et al., 2010;Cicmil et al., 2014;Moradi et al., 2003;Nasiotis et al., 2017;Perry et al., 2011), that is, estimating the neuronal sources (i.e., the pRFs) underlying the MEG sensor signals through inverse modeling. However, inverse modeling in MEG is a non-unique problem, as there is, without additional assumptions, an infinite number of source combinations that could lead to the same measured MEG signal. Moreover, spatial resolution is limited by field spread, also known as spatial leakage in source space (Wens, 2023). This makes it difficult to estimate receptive field maps with fine detail and previous studies could only distinguish pRF properties on the scale of whole visual quadrants or distinguish fovea versus peripheral pRFs (Brookes et al., 2010;Cicmil et al., 2014;Moradi et al., 2003;Nasiotis et al., 2017;Perry et al., 2011).

Kupers et al. (2021)introduced a forward modeling approach that avoids the inverse problem and instead uses the spatially-specific pRF properties obtained from fMRI measurements. By predicting the cortical responses and converting them to the MEG sensor level (instead of the other way around), they explained a large amount of variance in the MEG data. However, because Kupers and colleagues relied on stimulus-driven oscillations at 10 Hz, their approach does not readily provide access to MEG’s high time resolution.

Here, we extended the forward modeling approach to the temporal domain by adjusting the experimental design, analysis pipeline, and the type of recorded MEG signal. Specifically, we focused on event related fields (ERFs), that is, the brain’s response following a short stimulus event. Stimulus-driven changes in oscillations and ERFs are related measures of neural activity; however, they are not identical and differ in fundamental aspects (Bae & Luck, 2019;Schneider & Maguire, 2018;Yeung et al., 2004). Most importantly, ERFs reveal how brain activity evolves over time.

We show that for ERFs, the fMRI-based pRFs explain up to 91% of the measured MEG signal. Furthermore, we provide evidence that we can investigate pRF characteristics in detail by changing the pRF properties and quantifying how this affects the explained-variance of the ERF responses. Importantly, we do this with millisecond resolution, allowing us to further our understanding of both spatial and temporal characteristics of the visual system.

## 2 Methods

### 2.1 Participants

Five subjects (one female; ages 20–48 years, M = 29.8, SD = 9.9) participated in this study. Statistical power does not only depend on the number of participants but also on the number of trials per participant (Baker et al., 2021). We aimed to get significant results in individual participants by using a large number of trials per participant. In other words, participants are replication units not measurement units. Thus, this consists of a focused study (Gratton et al., 2022), with the aim to show significant results in every single participant. In addition, to have clinical relevance we need to get significant results in single participants. Lastly, the number of participants is similar as in previous modeling research using MEG (Ales et al., 2010;Brookes et al., 2010;Cicmil et al., 2014;Kupers et al., 2021;Moradi et al., 2003;Nasiotis et al., 2017;Perry et al., 2011) and fMRI (Aqil et al., 2021;Dumoulin & Wandell, 2008;Kay et al., 2013;Zuiderbaan et al., 2012).

All participants were screened for fMRI and MEG compatibility, had normal or corrected-to-normal visual acuity, and did not have a history of neurological or psychiatric disorders. The study was approved by the Scientific and Ethical Review Board (VCWE) of the Faculty of Behavior & Movement Sciences, Vrije Universiteit Amsterdam, and it was conducted in accordance with the ethical guidelines outlined in the Declaration of Helsinki (World Medical Association, 2000). All participants gave their written informed consent prior to the study.

### 2.2 Stimuli

Stimuli were created using the Python PsychoPy package (Peirce, 2007) and custom built Python code upon exptools2. Both fMRI and MEG stimuli were moving checkerboards presented inside apertures of different shapes on a gray mean-luminance background. The checkerboard inside the apertures consisted of multiple rows of squares. Each square had a width of 0.625deg of visual angle (deg). The rows of squares appeared to be moving either upwards or downwards, making a full cycle 5 times per second. This motion direction of the squares was chosen randomly for each stimulus presentation. To ensure alertness and stable fixation during the entire experiment, a small fixation dot with a diameter of 0.1deg was displayed in the middle of the screen, changing colors (red or green) in a semi-random interval with an average duration of 5.6 s. Participants were instructed to press a button whenever the color changed. The participants could decide to use the button with their left or right hand as the task and motion induced by the responses were unrelated to our experimental manipulation. We did not assess task performance, since it was not relevant to our analysis.

#### 2.2.1 Stimulus displays

##### 2.2.1.1 fMRI

Participants viewed the stimuli on an MRI-compatible screen (Cambridge Research System 32” LCD widescreen) located outside the bore through front-silvered mirrors. The screen resolution was 1920 x 1080 pixels with a 120 Hz refresh rate and height of 39.3 cm. The viewing distance was 210 cm yielding 10.68deg in diameter. Stimulus presentation was then limited to a circular area with the same diameter of 10.68deg.

##### 2.2.1.2 MEG

MEG stimuli were back projected via two mirrors onto a transparent screen using an LCD projector (BarcoData 8200 LC, Barco Projection Systems, Kuurne, Belgium) with a resolution of 1024 x 768 pixels and 60 Hz refresh rate, height of 34 cm, and viewing distance of 199 cm. The resulting circular window for stimulus presentation was 9.77deg in diameter.

#### 2.2.2 Stimulus and sequence

##### 2.2.2.1 fMRI

We used a standard contrast-defined bar-stimulus (Dumoulin & Wandell, 2008). The stimulus apertures for the fMRI session were bars with a width of 1.25deg. A bar was presented in one of eight possible configurations: four possible orientations (0, 45, 90, and 135deg) and two opposing step directions that were orthogonal to the bar orientation. The two opposing step directions were included to average out effects of step direction and avoid biasing the pRF estimates due to the lag of the hemodynamic response function (HRF). The bars swept across the 10.68deg circular area of the screen in 20 discrete steps. The presentation was time locked to each volume acquisition, so that each step was presented for 1.5 s. After every two bar passes, a mean-luminance screen was displayed for 15 s. Generally, six runs were acquired per participant. For participant 1, we had time to acquire eight runs within the scheduled scanning time. For participant 2, we acquired just four runs due to technical issues. Each run had a duration of about 6 min. The order of the eight bar configurations presentation was the same for each run. Together with preparation and structural scans, the fMRI session took about 1.5 h in total.

##### 2.2.2.2 MEG

We used the same contrast-defined stimulus, but the aperture was either a bar or a circle shape. The bars had a width of 1.25deg and were either vertically or horizontally oriented. They were presented at 0, 1.28, or 3.06deg away from the center of the screen (vertical bars: left/right from center; horizontal bars: below/above center). This yielded five possible locations per bar orientation. The circle shapes appeared in one of eight locations, that is, in one of the four quadrants with two possible eccentricities (1.28 and 3.06deg). Circles at the small and large eccentricity had a diameter of 1.25 and 2.5deg, respectively. The circle shapes were included to increase the spatial specificity of the signal by only stimulating one quadrant of the visual field at a given time. The bar- and circle-configurations yielded a total of 18 MEG stimuli, which were each presented 18 times per run.

In contrast to the fMRI sequence, the stimuli were presented in semi-randomized order, with the restriction that a given stimulus did not consecutively appear twice in the same visual quadrant. Furthermore, stimulus duration was short (100 ms) and followed by a mean-luminance screen (lasting 900 ms ± 250 ms) to evoke event-related field (ERF) responses.

The stimulus presentation protocol was interleaved with 2 s blink breaks about every 20 s to minimize blink artifacts in the MEG data. Blink breaks were indicated by a black square in the middle of the screen, and the participant was instructed to try to blink only during this interval. The participant performed the same fixation dot color change task as during the fMRI session; however, color changes did not appear during stimulus presentation or blink breaks to leave them uninterrupted. The participant could take breaks in between runs (while staying in the scanner). Per participant, ten runs were recorded that each lasted about 6 min. For participant 1, nine runs were recorded due to time constraints, and for participant 2 fourteen runs could be collected in the scheduled scanning time. Including preparation, the scanning session took about 2 h.

### 2.3 Data acquisition

#### 2.3.1 fMRI

Structural and functional MRI data was acquired using a Philips Achieva 7T scanner with a 32-channel Nova Medical head coil. For structural whole-brain images, T1-weighted (T1w) and T2-weighted scans were acquired with a resolution of 0.7 mm isotropic. Functional MRI data were acquired using a T2*-weighted 2D-EPI sequence with a resolution of 1.7 mm isotropic and 57 slices and 225 volumes (FOV = 216 x 216 mm^2^, TR = 1500 ms, TE = 2 ms, FA = 53deg, duration = 330 s). To avoid start-up magnetization transients, the first 9 s were automatically discarded (six dummy scans). Top-up scans with opposite phase-encoding direction were acquired after each scan to perform susceptibility distortion correction (Andersson et al., 2003).

#### 2.3.2 MEG

MEG data were recorded in a magnetically shielded room using a 306-channel whole-head Triux Neo system (Elekta Neuromag Oy, Helsinki, Finland) of which 102 sensors were magnetometers and 204 planar gradiometers. The sampling rate was 1000 Hz and during the recording, the data were low-pass filtered at 330 Hz to avoid aliasing and high-pass filtered at 0.10 Hz. Internal active shielding (IAS;Taulu et al., 2014), using MEGIN’s in-wall feedback-coils, was used. The gantry of the MEG was angled at 69deg, and the participants were sitting in an upright position. Five head localization coils were used to determine the head position relative to the MEG sensors throughout the recording session. The position of these coils and points outlining the scalp and nose was digitized using a 3D-digitizer (Fastrak, Polhemus, Colchester, VT, USA) before the recording. For post hoc removal of artifacts, the electrooculogram (EOG; with three electrodes, two placed laterally to both eyes and one above the left eyebrow) and electrocardiogram (ECG; one electrode) were recorded. During initial data preprocessing, we compared ERF time courses with and without excluding artifact-affected epochs and concluded that rejection of the affected epochs did not result in significantly different ERF profiles. Therefore, we did not use EOG and ECG for artifact rejection. For precise epoching based on stimulus onsets, a photodiode was placed in the corner of the screen measuring the exact onset and offset of the stimuli.

### 2.4 Data preprocessing

#### 2.4.1 fMRI

For structural MRI data, FreeSurfer (version 7.2.0)*recon-all*was used to obtain native cortical surface reconstructions (‘fsnative’ surfaces). Functional MRI data preprocessing was performed using the standard FMRIPREP pipeline (version 20.2.0;Esteban et al., 2019), a Nipype-based tool (Gorgolewski et al., 2011). Each T1w (T1-weighted) volume was corrected for INU (intensity non-uniformity) using*N4BiasFieldCorrection*(version 2.1.0;Tustison et al., 2010) and skull-stripped using*antsBrainExtraction.sh*(version 2.1.0; using the OASIS template). Functional data was slice time corrected using*3dTshift*from AFNI (version 16.2.07;Cox, 1996) and motion corrected using*mcflirt*(FSL version 5.0.9;Jenkinson et al., 2002). Distortion correction was performed using an implementation of the TOPUP technique (Andersson et al., 2003) using*3dQwarp*(AFNI version 16.2.07;Cox, 1996). This was followed by co-registration to the corresponding T1w using boundary-based registration (Greve & Fischl, 2009) with 6deg of freedom, using*flirt*(FSL). Motion correcting transformations, field distortion correcting warp, BOLD-T1w transformation and T1w-to-template (MNI) warp were concatenated and applied in a single step using*antsApplyTransforms*(ANTs version 2.1.0) using Lanczos interpolation. Physiological noise regressors were extracted applying CompCor (Behzadi et al., 2007). Principal components were estimated for the two CompCor variants: temporal (tCompCor) and anatomical (aCompCor). A mask to exclude signal with cortical origin was obtained by eroding the brain mask, ensuring it only contained subcortical structures. Six tCompCor components were then calculated including only the top 5% variable voxels within that subcortical mask. For aCompCor, six components were calculated within the intersection of the subcortical mask and the union of CSF and WM masks calculated in T1w space, after their projection to the native space of each functional run. Frame-wise displacement (Power et al., 2014) was calculated for each functional run using the implementation of Nipype. Many internal operations of FMRIPREP use Nilearn (Abraham et al., 2014), principally within the BOLD-processing workflow. For more details of the pipeline seehttps://fmriprep.readthedocs.io/en/20.2.0/workflows.html. This description was generated by FMRIPREP intended to be copied verbatim (released under the CC0 license), to enhance reproducibility of studies. We converted the preprocessed functional time series (resampled in*fsnative*surface space) to percent signal change, which was a matrix of shape vertices x time for each run. The number of vertices across participants ranged from 275064 to 617224, with a mean of 370937.

#### 2.4.2 MEG

To remove environmental artifacts and because IAS was used (Taulu et al., 2014), the raw MEG data werefirst preprocessed with the temporal extension of Signal Space Separation (tSSS;Taulu & Simola, 2006) implemented in the MaxFilter software (Elekta Neuromag, Oy, version 2.2.15). Malfunctioning or noisy channels during the entire session were interpolated by tSSS (an average of 5.5 out of 306 channels were interpolated). The data files were then read with MNE (Gramfort et al., 2013;Larson et al., 2022) and subsequently further preprocessed using a custom-written Python code. The photodiode recorded alongside the MEG data was used to identify onsets of stimuli, and periods containing missed screen flips were discarded. The data were then epoched with a time window of -100 to 600 ms from stimulus onset. The procedure left on average 186.2 epochs per stimulus. Every epoch was then baseline corrected, subtracting the mean amplitude during the baseline period (-100 to 0 ms from stimulus onset) from the entire epoch. No other preprocessing or filtering steps were applied.

#### 2.4.3 Gain matrix

The gain matrix, otherwise also referred to as the lead field matrix, describes how each cortical source (pRF that we measured with fMRI) contributes to the signal measured at each MEG sensor, given the anatomy of the participant and the location of their head with respect to the sensors. In other words, the folding influences the MEG signals (Ales et al., 2010;Hillebrand & Barnes, 2002). We used Brainstorm (Tadel et al., 2011) to co-register the MEG data to the participant’s MRI anatomy and then compute the gain matrix. For first gross alignment of the MEG data to the anatomy, we defined the participant’s fiducials on the participant’s T1w image. Brainstorm then refined the registration using the digitized head points and the scalp surface extracted from the T1w image. Some manual adjustments were made when necessary. Once aligned, we used the Overlapping Spheres algorithm (Huang et al., 1999) implemented in the Brainstorm toolbox to generate the gain matrix, for which for each sensor separately a local sphere is fitted to the participant’s skull-shape below the sensor. The FreeSurfer-generated pial surface for each participant was used as source-space, and an equivalent current dipole was placed at each vertex. We constrained the orientation of the dipoles to be perpendicular to the surface. The resulting gain matrix was of shape sensors x vertices.

### 2.5 Analysis pipeline

We used a forward modeling approach to estimate pRFs with high spatial and temporal resolution. To outline briefly, we measured fMRI and MEG in separate sessions while showing contrast-defined stimuli to five participants. With the measured fMRI response, we estimated the participants’ pRFs on their cortical surface (Fig. 3). These pRFs were used to predict their cortical responses to the MEG stimuli. The predicted responses were then converted to sensor space by applying a gain matrix, which describes how each cortical location contributes to the signal measured with the MEG sensors. We then compared the predicted sensor responses to the measured sensor responses at each timepoint recorded around stimulus presentation. With this approach, we were able to investigate with millisecond resolution how well the pRFs describe the MEG signal. Below, we explain the individual steps in more detail.

#### 2.5.1 Estimate pRF properties with fMRI

We measured fMRI while participants viewed contrast-defined bar stimuli that traversed across the screen. Based on measured fMRI response, we estimated the participant’s pRFs as described inDumoulin and Wandell (2008). This was done for each location on their cortical surface (referred to as ‘vertex’). In short, we extrapolated the measured fMRI response of each voxel to the vertices on the participant’s cortical surface. In a two-stage coarse-to-fine search, we then constructed a range of 2D Gaussian models with different visual field locations and sizes and predicted their responses to the stimuli presented during the fMRI session. This was done by multiplying the 2D Gaussian model with the stimuli and convolving the resulting time course with a hemodynamic response function (HRF). The HRF was modeled as two gamma basis functions (Glover, 1999) with its coefficients set to 1, 1, and 0 for the canonical HRF, its derivative and dispersion, respectively (Pedregosa et al., 2015). Each predicted response was then compared to each vertex’ measured response. The pRF (i.e., 2D Gaussian) that best predicted the response was then used as a seed to fine-tune the preferred pRF position and size that fitted the vertex’ fMRI response the best. For each cortical location, we now had an estimate of the part of the visual field that each vertex responds to the most, that is, its preferred population receptive field.

For the remainder of the analysis, we only considered vertices that were responsive to visual stimuli and could be reliably estimated (Fig. 3). In other words, a vertex was included if (i) the vertex was not located under a vein, (ii) the pRF explained at least 15% of the measured fMRI response, and (iii) the pRFs’ position in visual space was inside the circular aperture in which the stimuli were presented.

#### 2.5.2 Measure ERF sensor responses with MEG

In a separate session, we measured MEG with 306 sensors while showing 18 bar and circle stimuli at different visual field locations. In contrast to the fMRI experiment, the stimuli were presented for a shorter duration (100 ms) and in semi-random order; however, the checkerboard pattern within the circle and bar stimuli was kept the same as in the fMRI experiment with the aim to elicit strong responses in equivalent neuronal populations as in the fMRI experiment. Each stimulus duration was followed by a blank period of about 900 ms (with a jittered onset of ± 250 ms) to allow for the neuronal responses to go back to baseline. We calculated the event-related field (ERF) responses to the 18 stimuli by averaging the epochs from -100 to 600 ms after stimulus presentation for each stimulus type separately and for each sensor independently. For visualization of the ERFs inFigure 4B, we averaged over all epochs of a given stimulus. However, for cross-validation in our analysis, we calculated the ERF averages over a random selection of half of the epochs per stimulus for a given cross-validation ‘fold’, resulting in a ‘training’ and ‘test’ ERF data set. We did this 120 times, resulting in 120 ‘training’ and ‘test’ ERF data sets.

#### 2.5.3 Predict cortical responses from pRFs

Next, for each pRF on the participant’s cortical surface, we predicted their responses to the same 18 MEG stimuli. The predicted value for each stimulus is the overlap between the 2D pRF model and the 2D binarized stimulus, both living in visual space. We calculated the overlap as the matrix multiplication of the vertices’ pRFs and each of the stimuli, resulting in a matrix of shape vertices x stimuli. Each vertex has thus one predicted value per stimulus, which is zero if there is no overlap, low if the overlap is small, and high if the overlap of stimulus and pRF is large.

#### 2.5.4 Predict ERF sensor responses

The ERF signal measured with MEG resides at the sensor level, whereas the pRF predictions originate from the participant’s cortical surface. To be able to test how much the pRF models explain the ERF signal, we first needed to convert the pRF predictions to the sensor level using the gain matrix. The gain matrix contains the weights explaining how activity at each vertex on the cortical surface translates to a magnetic signal in each sensor. We obtained the predicted sensor responses by matrix multiplication of the gain matrix (sensors x vertices) with the pRF predictions (vertices x stimuli), resulting in a matrix of size sensors x stimuli containing each MEG sensor’s prediction to the 18 stimuli.

#### 2.5.5 Compare measured and predicted sensor responses

We then tested how well the pRF models explained the measured ERF responses at each latency. For that, we fitted each sensor’s predicted responses to the measured ERF responses at each timepoint between -100 and 600 ms after stimulus onset in a cross-validated manner (i.e., for the 120 sets of randomly split training and test ERF halves that we call cross-validation ‘folds’).

The following provides a more detailed explanation. For a given cross-validation fold, latency, and sensor, we obtained two beta weights, b1 (scaling factor) and b0 (offset), by linearly regressing the pRF predictions of a given sensor,p(sensor), on an ERF training set,trainingY(fold,latency,sensor). Since we baseline corrected the epochs, we kept the offset fixed at 0 and only used the scaling factor to scale the pRF predictions ($\hat{Y}$):

$$\begin{array}{l} \hat{Y}\left(fold,latency,sensor\right)=b1\left(fold,latency,sensor\right)*\\ \;\;\;\;\;\;\;\;\;\;\;\;\;\;\;\;\;\;\;\;\;\;\;\;\;\;\;\;\;\;\;\;\;\;\;\;\;\;\;\;\;\;\;\;\;\;\;\;\;\;\;\;\;\;\;\;\;\;\;\;\;\;\;\;\;\;\;\;\;\;\;\;\;\;\;\;p\left(sensor\right) \end{array}$$ (1)

This brought the pRF predictions to the same units as the ERF responses (femto Tesla (fT) for magnetometers and fT/m for planar gradiometers). We now computed the goodness of fit of the scaled pRF predictions ($\hat{Y}(fold,$latency,sensor)) on a test ERF data set (testY(fold,latency,sensor)) using variance explained (VE):

$$VE\left(fold,latency,sensor\right)=1-\left(\frac{RSS\left(fold,latency,sensor\right)}{TSS\left(fold,latency,sensor\right)}\right)$$ (2)

where the Residual Sum of Squares (*RSS*) and Total Sum of Squares (*TSS*) were defined as:

$$\begin{array}{l} RSS\left(fold,latency,sensor\right)\\ =\sum_{stimulus}(testY_{stimulus}\left(fold,latency,sensor\right)\\ {\;\;\;-{\hat{Y}}_{stimulus}\left(fold,latency,sensor\right))}^{2} \end{array}$$ (3)

and

$$\begin{array}{l} TSS\left(fold,latency,sensor\right)\\ \;\;\;\;=\sum_{stimulus}{\left(testY_{stimulus}\left(fold,latency,sensor\right)\right)}^{2} \end{array}$$ (4)

*VE*is thus a single value that encompassed how much the predicted pRF responses explained the measured ERF responses to the 18 stimuli at a particular timepoint, in a given sensor, and for a given fold. In theresultssection, we reported the median variance explained over the 120 folds, together with its 95% confidence interval (CI).

To obtain sensor averages, we did not directly average over the sensors’VE, since the sensors differ in their time to peak. Instead, we calculated theaverageVEfor a given fold, timepoint, and group of sensors as:

$$\begin{array}{l} averageVE\left(fold,latency\right)\\ \;\;\;\;=1-\left(\frac{\sum_{sensor}RSS_{sensor}\left(fold,latency\right)}{\sum_{sensor}TSS_{sensor}\left(fold,latency\right)}\right) \end{array}$$ (5)

We again reported the median and CI over cross-validation folds. We used three different sensor groups to obtain the averages. For the first group, we calculated the average across all sensors that had reached a variance explained of 50% anywhere between -100 and 600 ms of stimulus presentation (‘active sensors’). For the second group, we averaged over all sensors with variance explained higher than 50% in an early time window from 50 to 150 ms after stimulus onset (‘early sensors’). And for the third group, we averaged over the 25% fastest sensors, that is, the 76 sensors that reached 50% variance explained earliest after stimulus onset (‘earliest sensors’).

#### 2.5.6 Shift pRF polar angles and re-fit

To test whether the recorded ERFs were optimally described by the pRF parameters as estimated by fMRI, we shifted the polar angles of each cortical pRF away from its original polar angle by a set of negative and positive angles (-90, -60, -45, -30, 0, 30, 45, 60, and 90deg) and repeated the analyses described inSections 2.5.3to2.5.5. We hereby obtained a new set of sensor predictions based on the*shifted*pRFs and compared them again to the experimental ERF data. We expected that if the ERF responses are driven by the cortical pRFs, the variance explained should decrease as a result of shifting the pRFs away from their estimated parameters.

## 3 Results

### 3.1 pRF models explain ERF responses across different stimuli

**Fig. 1.**
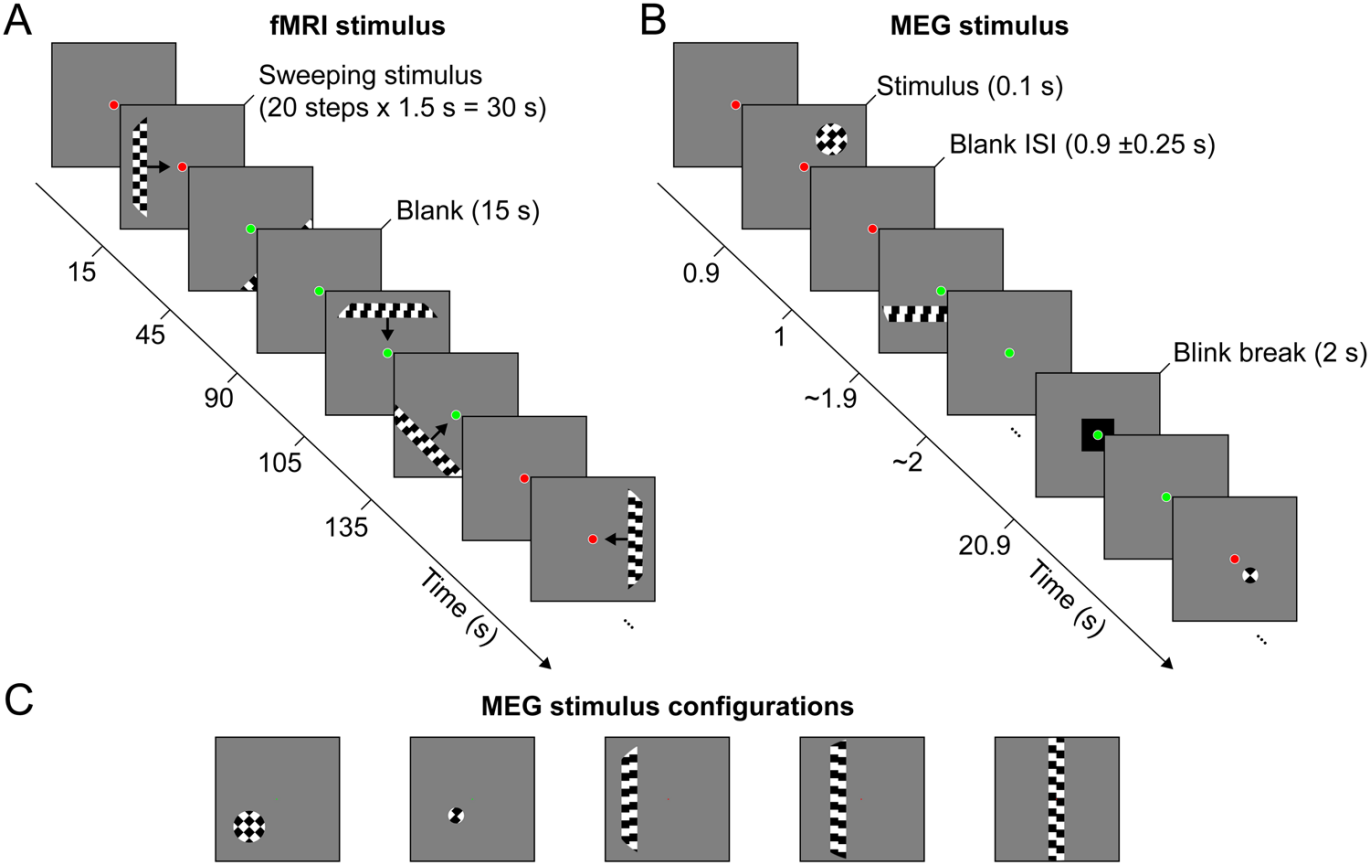
Experimental design. (A) For the fMRI session, we used a standard stimulus sequence to estimate the participant’s pRFs (Dumoulin & Wandell, 2008). A contrast-defined bar swept across the screen in eight different directions interspersed with mean-luminance blocks. The step direction was orthogonal to the bar orientation. (B) During the MEG session, 18 contrast-defined circle or bar stimuli were presented for 100 ms and interspersed by mean-luminance breaks (900 ± 250 ms). (C) MEG stimulus configurations with example contrast-defined circle and bar stimuli. The figure illustrates the two possible eccentricities of the circle stimuli (1.28 and 3.06deg from screen center) in one of the four visual quadrants in which the circles could appear. For the bar stimuli, the figure shows the vertical bar at three out of five possible eccentricities (0, 1.28, and 3.06deg from screen center). The protocol also included five horizontal bars.

We presented 18 different stimuli to the participants (Figs. 1Cand4A) while measuring MEG to obtain ERF responses.Figure 4B(right panel) shows a participant’s example sensor ERF responses to the 18 stimuli. The stimuli evoked clear ERFs as commonly reported in literature (e.g.,Hashimoto et al., 1999;Parker et al., 1982;Tabuchi et al., 2002). The ERFs were spatially specific, that is, stimuli at different spatial locations evoked different ERFs.

**Fig. 2.**
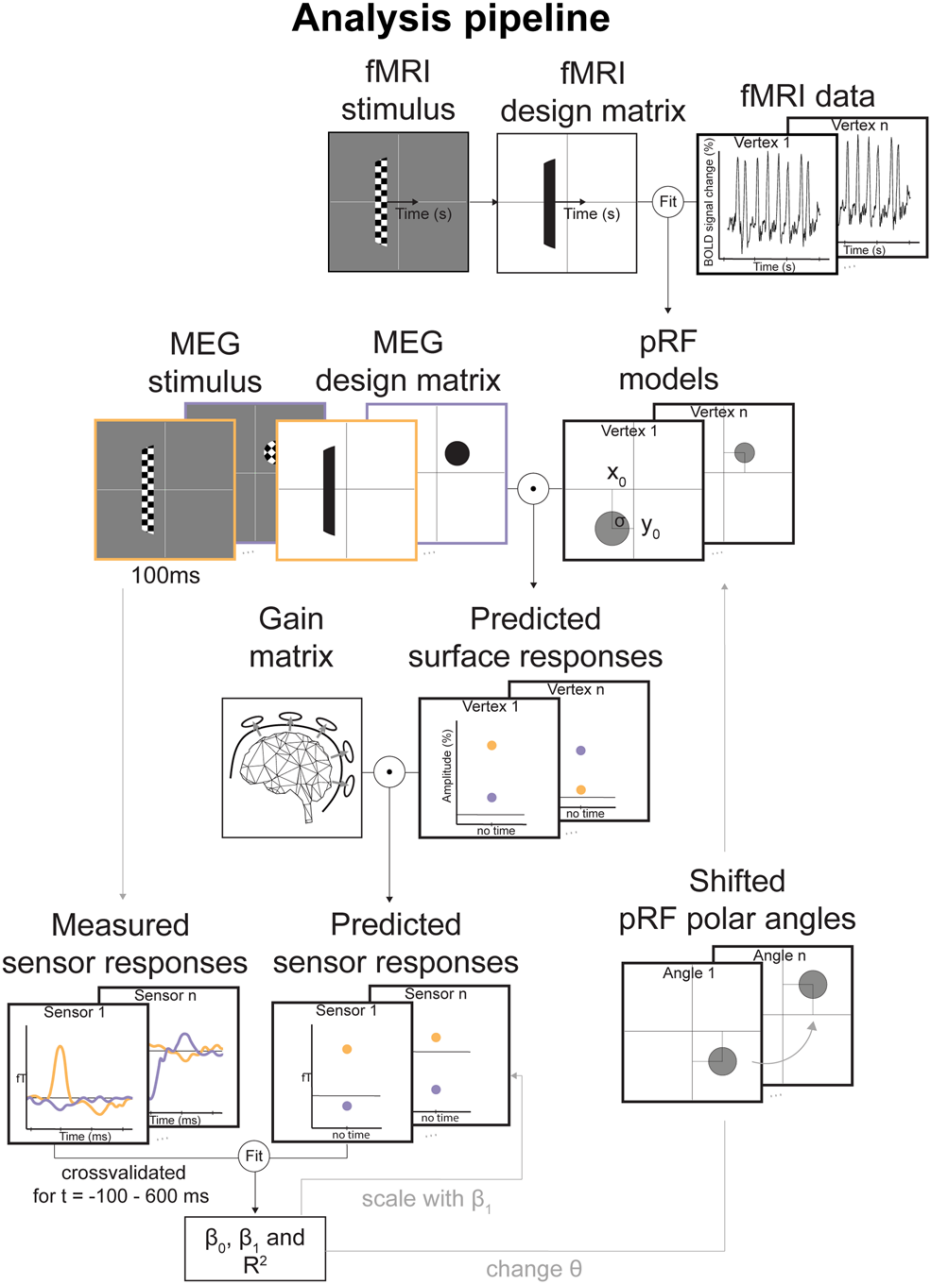
Analysis pipeline.*Step 1) Estimate pRF properties*: We measured fMRI responses while the participants viewed contrast-defined bar stimuli that traversed across the screen to obtain pRF estimates for each location on the participant’s cortical surface (Dumoulin & Wandell, 2008).*Step 2) Measure ERF responses*: In a separate session, we measured ERF responses with MEG while the participants viewed contrast-defined bar or circle stimuli. We obtained ERF responses by averaging over the stimulus epochs per stimulus type separately.*Step 3) Predict cortical responses from pRFs*: Next, we used the pRF models to predict responses to the MEG stimuli, obtaining 18 predicted stimulus values for each cortical location (‘predicted surface responses’).*Step 4) Predict ERF sensor responses*: Since the ERF responses were measured in the MEG sensors, we converted the cortical pRF responses to the sensors by a matrix multiplication of the gain matrix (sensors x vertices) with the surface responses (vertices x stimuli). The gain matrix indicates how much each cortical location contributes to a given sensor (Huang et al., 1999).*Step 5) Compare measured and predicted sensor responses*: For each timepoint between -100 and 600 ms after stimulus onset, we compared each sensor’s pRF predictions to the ERF responses of the same sensor at each timepoint using cross-validated linear regression.*Step 6) Shift pRF polar angles and re-fit*: To evaluate whether the recorded ERFs were optimally fitted by the pRF parameters as estimated by fMRI, we shifted the pRF polar angles between -90 and 90deg in separate fitting procedures and repeated steps 3 to 5. We expected the explained variance to drop compared to the explained variance for the original pRF polar angle.

Next, we evaluated whether the pRFs measured with fMRI could capture these ERF responses. In brief, we used the participant’s pRF models to predict each sensor’s responses to the same 18 MEG stimuli (Fig. 4Bleft panel,Fig. 2steps 3 and 4) and computed how much the pRF prediction explained the ERF signal at any given timepoint around stimulus presentation, using cross-validated variance explained as a measure of goodness of fit. At stimulus onset (t = 0 ms), the pRFs explained 0% of the ERF responses (Fig. 4C). This is not surprising as no visual evoked signals are present. For this sensor, the maximum variance explained by the pRF models for these ERF signals was 75.6%. Most variance was explained between 75 and 250 ms (Fig. 4D; i.e., CI was above 0) with a drop in variance explained around 150 ms, corresponding to a decrease in ERF responses at that time. Note that the variance explained can become negative (Fig. 4D; i.e., CI below 0 before 75 ms and after 300 ms) due to the cross-validation procedure in which variance explained is bounded by -infinity and 1 (seeSupplementary Fig. 1).

**Fig. 3.**
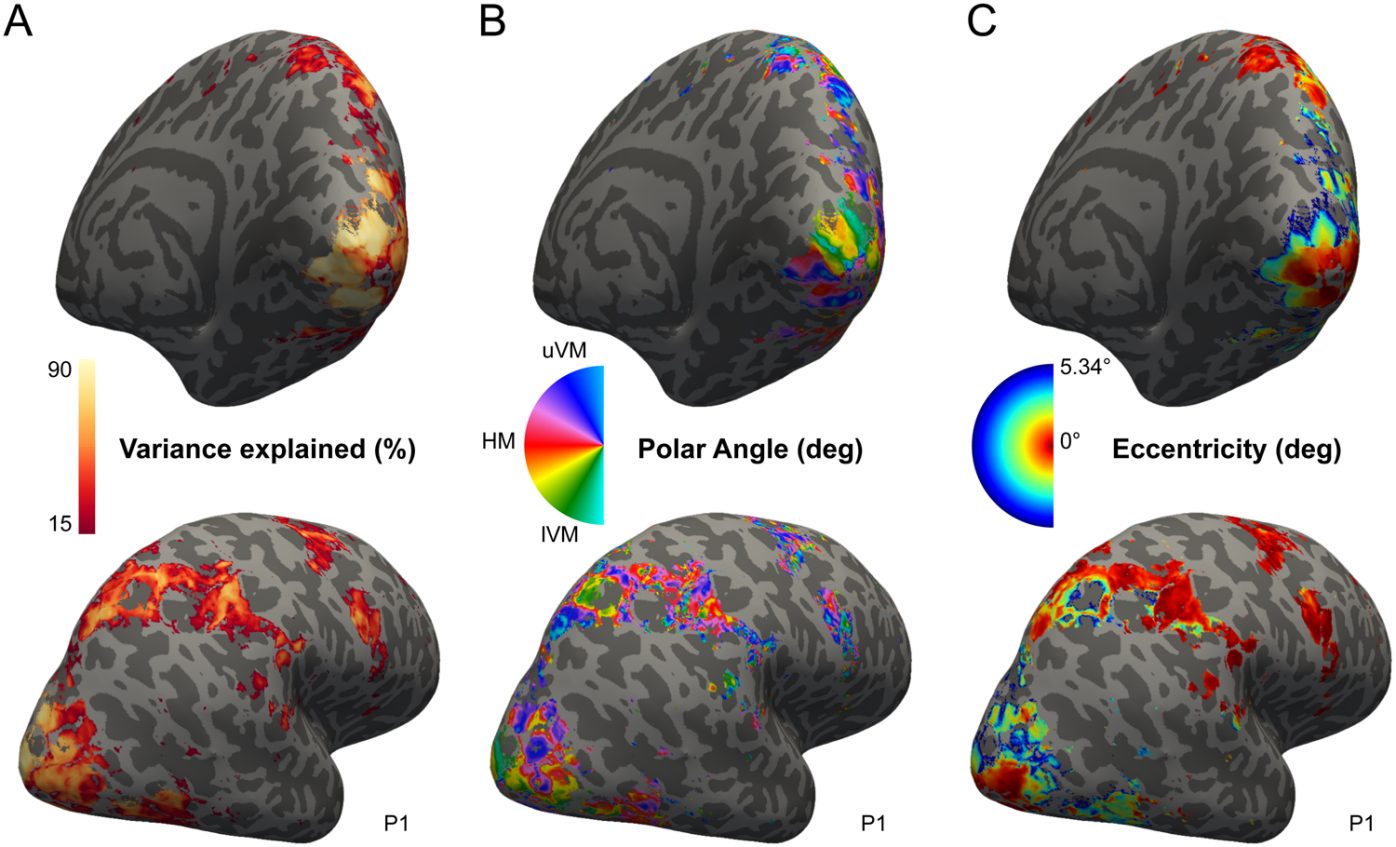
fMRI pRFs and visual field maps. Right hemisphere of participant 1, displaying pRFs properties of the cortical locations (vertices) included in the analysis. The top row shows a medial view, and the bottom row shows a lateral view. The colored vertices show: (A) how much variance a given pRF explained in the fMRI data (r^2^); (B) the polar angle in visual space, ranging from the upper vertical meridian (uVM) through the horizontal meridian (HM) to the lower vertical meridian (lVM); (C) eccentricity.

**Fig. 4.**
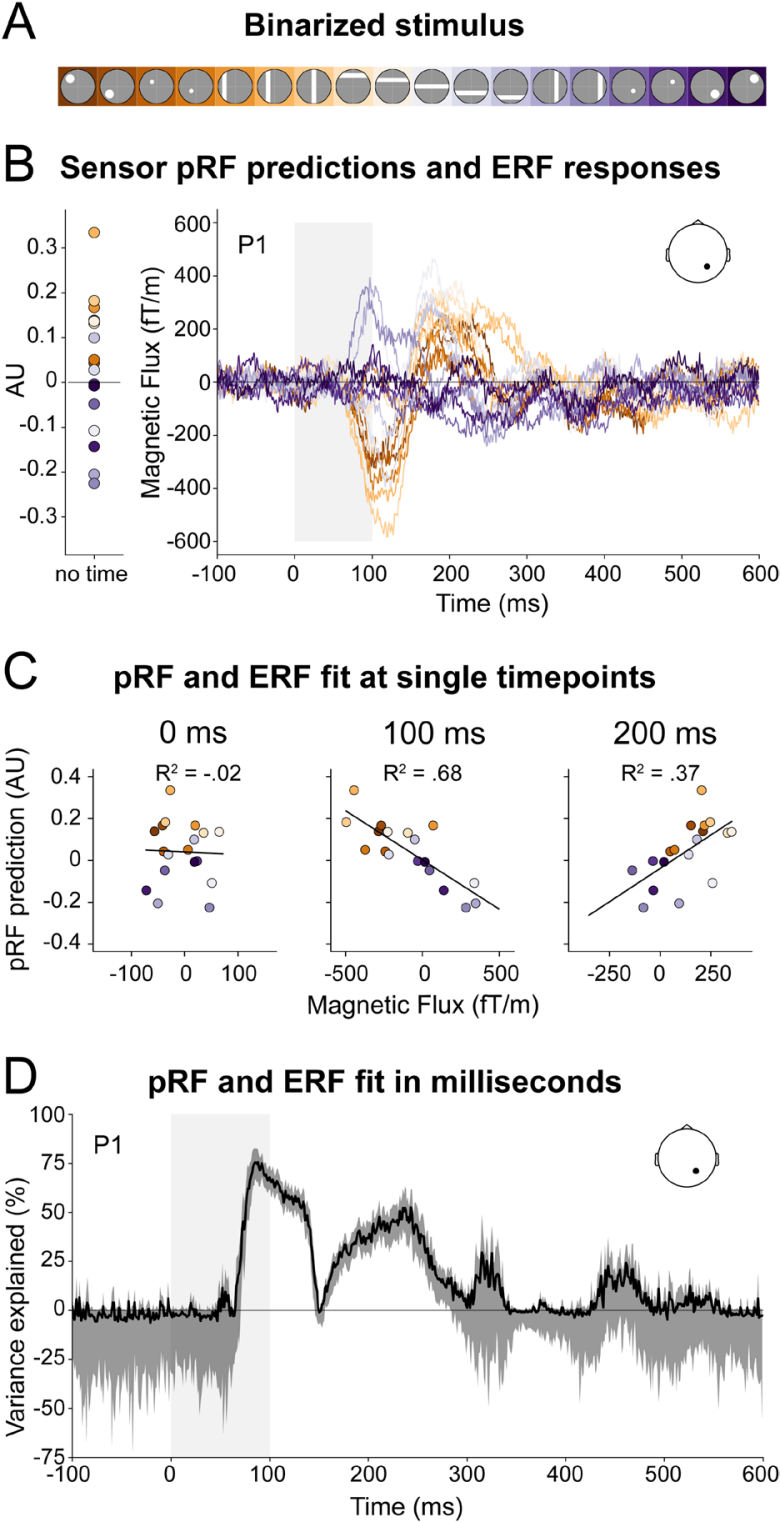
pRF-ERF fit for an example sensor. (A) The 18 stimuli apertures color coded according to visual field location, from left (orange) to right (purple). (B)*Left*: One sensor’s pRF predicted responses to the 18 stimuli (color coded as in A) for participant 1. The pRF models predicted different responses to the different stimuli.*Right*: The sensor’s ERF responses to the same stimuli. The sensor’s location is marked as the dot on the head layout. Different stimuli elicited different ERF responses. The gray box marks the duration of the stimulus. (C) Fits between the sensor’s ERF responses and pRF predictions for the different stimuli at three time points. The pRF predictions explained around 0%, 68%, and 37% of the variance of the ERF responses (Eq. 2) at timepoint 0 ms (stimulus onset), 100 ms, and 200 ms, respectively. (D) The amount of variance explained for the sensor’s ERF responses at each timepoint between -100 to 600 ms after stimulus onset. The black line and gray regions indicate the median variance explained and 95% confidence interval across the 120 folds of the cross-validation procedure, respectively. For this sensor, the pRFs explained the ERF responses between 75 to 250 ms.

### 3.2 pRF models explain ERF responses across many sensors

**Fig. 5.**
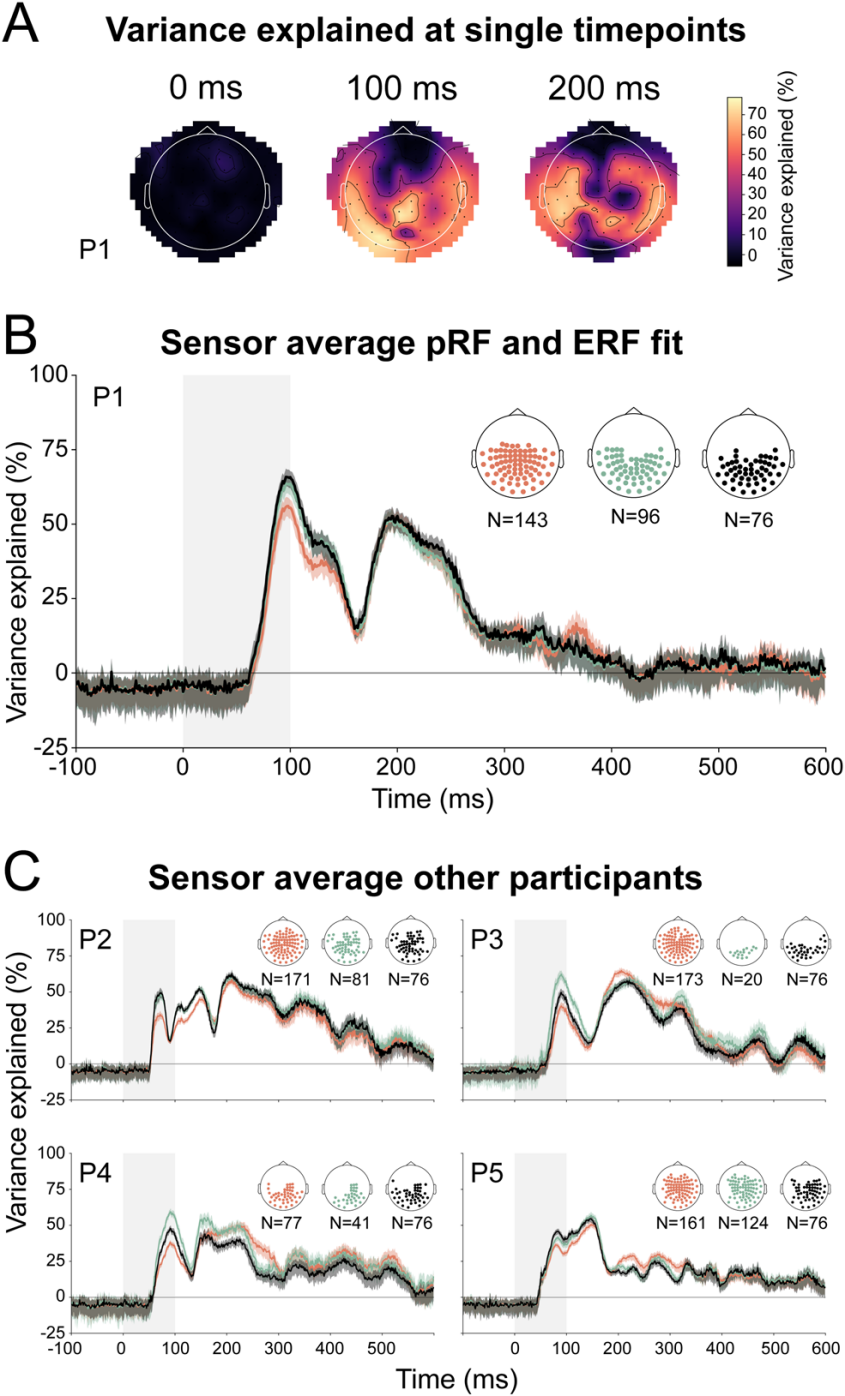
pRF-ERF fit across sensors. (A) Scalp distribution of variance explained for the 102 magnetometer sensors at three example timepoints (0, 100, and 200 ms after stimulus onset). The 204 planar gradiometers are shown inSupplementary Figure 2B & C. The white outline marks the participant’s head orientation with respect to the MEG helmet. (B) Mean variance explained time courses over three subsets of sensors (orange: ‘active sensors’, green: ‘early sensors’, black: ‘earliest sensors’). All sensor selections showed similar average variance explained after stimulus onset. (C) Sensor averages for the other participants. The exact shapes and timing of fits differed per participant; however, we observed the same overall trend where the pRFs capture the MEG signals between 75 and 250 ms.

The cortical pRF models explained the ERF responses across many sensors (Fig. 5A). We did not quantify the scalp distribution across time, because, ultimately, it is the cortical distribution that matters, not scalp distributions. Nevertheless, the scalp distributions illustrate that the pRF models explain the ERF responses across many MEG sensors.

Next, we averaged three different sensor selections, which resulted in similar variance explained time courses (Fig. 5B). The maximum single-sensor variance explained in the first participant was 86.7% at 188 ms.

We found similar trends for the other participants (Fig. 5C,Supplementary Fig. 3). For all participants, we saw a sharp rise around 75 ms after stimulus onset, two or more peaks of variance explained, and a decline of variance explained at about 250 ms after stimulus onset. Maximum single-sensor variance explained during the first sharp rise between 50 and 150 ms was between 84.8% and 90.6% across participants. The maximum variance explained for the second time window between 150 and 250 ms was between 86.7% and 91.0%. We show that by using the individual participants’ pRFs, we can explain individual ERFs we measured with MEG with high precision over time.

### 3.3 Altering pRF polar angles reduces the ability to explain ERF responses

**Fig. 6.**
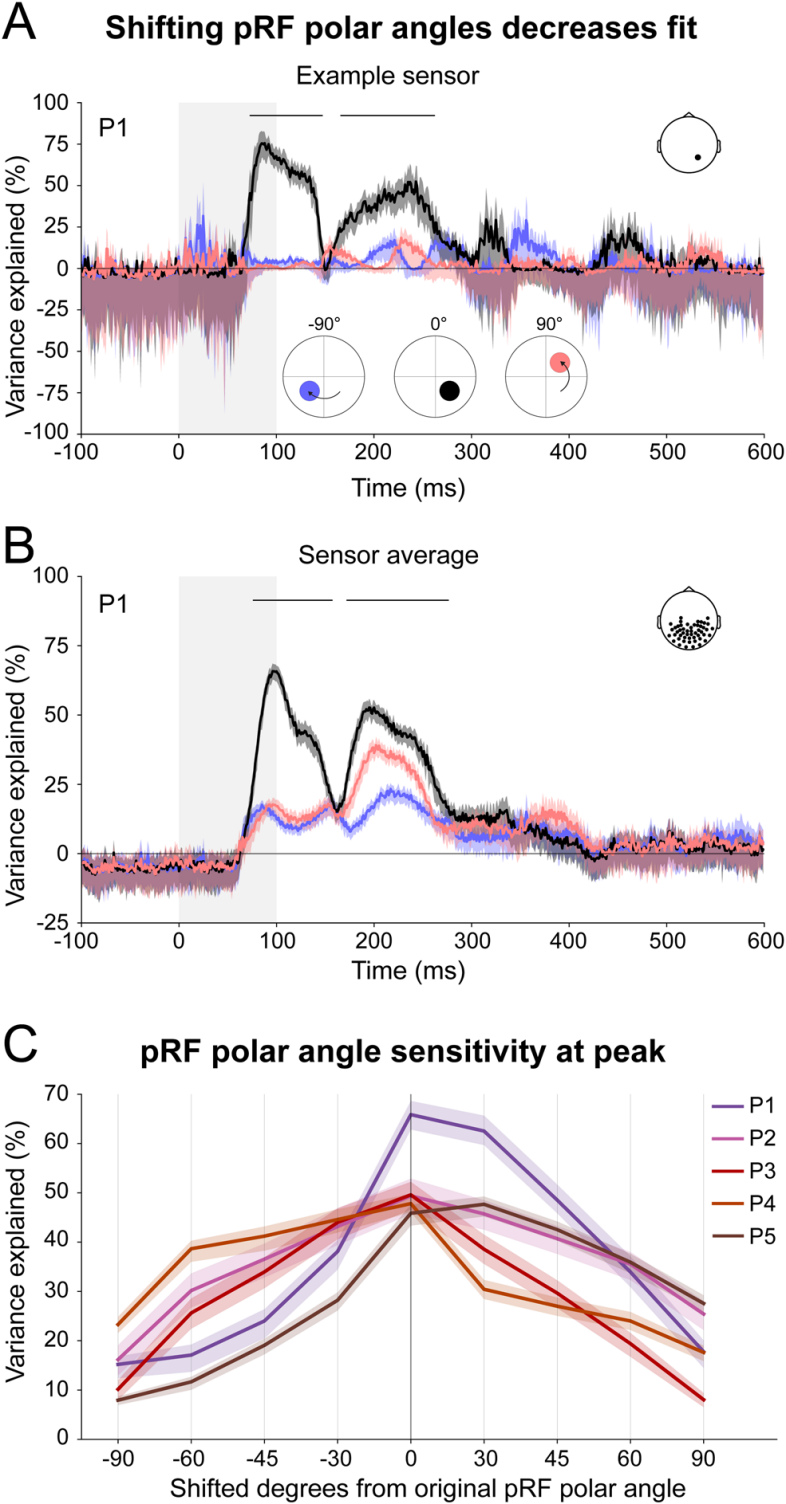
Sensitivity to pRF polar angles. (A) Fit of example sensor’s ERF values and the original pRF predictions (black line). The blue and pink show the fit of the pRFs that were shifted by 90deg clockwise and counterclockwise. Solid lines and fills indicate the medians and 95% confidence intervals over cross-validation folds. The variance explained decreased when the pRF positions were shifted away from the fMRI-estimated positions. Horizontal black lines on top mark the latencies where the fits were significantly decreased, that is, the CI of the shifted pRF fits were not overlapping with the CI of the original fit. (B) The average fit for ‘earliest sensors’ in the same participant (same ‘earliest sensors’ selection as inFig. 5B). We observed a significant decline of variance explained. (C) Variance explained for the ‘earliest sensors’ average across all shifted pRF polar angles, for all participants. Solid lines and fills correspond to each participant’s variance explained and CI at the timepoint of maximum variance explained for the 0deg shifted pRF. Variance explained declines when shifting the pRF polar angle away from the original pRF location.

We shifted each cortical pRFs’ away from its estimated polar angle to assess whether the ERFs were most effectively described by the pRFs polar angles as estimated by fMRI. We found that moving the pRF polar angles away from their initial location (black line inFig. 6A) resulted in a reduction of variance explained (blue and pink lines inFig. 6A). The horizontal black lines indicate at which latencies the fit of the altered pRFs were significantly decreased as compared to the original pRF polar angles, that is, the confidence intervals were not overlapping. We found a similar result for the sensor average of the ‘earliest sensors’ (Fig. 6B) across all participants (Fig. 6BandSupplementary Fig. 4). Thus, ERF responses were best explained by the fMRI-estimated pRFs, suggesting our method is sensitive to the pRF properties.

## 4 Discussion

We introduced a forward modeling approach that explains measured MEG signals with millisecond resolution. The participants’ pRFs were estimated on the cortical surface (Dumoulin & Wandell, 2008) and used to predict event-related responses we measured with MEG. We compared the predicted sensor responses to the measured ERFs, and our results showed that the pRF models explained the MEG sensor signal with millisecond resolution across sensors and participants (Figs. 4and5). Furthermore, through a perturbation-analysis we showed that the properties of the estimated neuronal population are important in explaining the signal (Fig. 6), demonstrating the utility of this forward modeling approach to reveal neuronal mechanisms with both high spatial and temporal resolution.

Our approach builds on our previous work described inKupers et al. (2021). Kupers and colleagues demonstrated that pRFs can capture stimulus-driven oscillations as measured with MEG. However, stimulus-driven oscillations lack high temporal resolution. Extending the method to ERF responses allowed us to investigate neuronal dynamics over time. We made several alterations to the original method: (i) we adjusted the experimental design to present the contrast-defined stimuli for 100 ms instead of 1.3 s. The short stimulus presentation allowed us to examine stimulus-evoked ERF responses, as compared to stimulus-driven oscillations. As a result, our temporal resolution was only limited by the sampling rate of the MEG scanner, which was 1000 Hz here; (ii) we adjusted the fitting procedure accordingly to accommodate the time domain and were able to evaluate the pRFs model performance with millisecond resolution.

In principle, we can estimate the responses to any visual stimuli. However, pRF properties are influenced by the stimulus layout (Dumoulin et al., 2014;Prabhakaran et al., 2020;Yildirim et al., 2018) and task (Klein et al., 2014;Womelsdorf et al., 2006). Essentially, the stimulus may elicit responses from different neuronal populations, whereas the task may elicit different interactions across the visual hierarchy. Therefore, we opted to keep the stimuli and task between the fMRI and MEG designs as similar as possible.

Other studies have investigated retinotopy with MEG using inverse modeling approaches (Brookes et al., 2010;Cicmil et al., 2014;Moradi et al., 2003;Perry et al., 2011). Inverse modeling aims to estimate the sources that produced the MEG signals measured at the sensor level. However, in contrast to our method that employs forward modeling only, inverse modeling comes with certain limitations. Inverse modeling can suffer from signal cancellation in the MEG sensors due to dipoles oriented in opposite directions (Nasiotis et al., 2017) and sources being radially oriented (Hillebrand & Barnes, 2002). While the neuronal activity from these sources cannot be estimated using inverse modeling since their signal is not picked up by the MEG sensors, our approach does not suffer from this limitation directly. Since our sensor predictions are informed by the gain matrix, which tells us which sources do not contribute to a given sensor, our predictions take the cancellation into account and thus partake in explaining the measured sensor signal.

Another limitation of inverse modeling is that the estimation problem is non-unique. There are an infinite number of possible source combinations that could lead to the measured MEG sensor signal and prior assumptions are needed to limit the potential solutions. As a consequence, previous MEG studies were able to capture well-established retinotopic characteristics, such as polar angle and eccentricity, only on the coarse scale of visual quadrants or distinguish foveal versus peripheral visual field. Our forward model, in contrast, is directly informed by the detailed map we obtained from the fMRI measurements, which has millimeter resolution. By changing the properties in a forward manner, we could quantitatively examine*which*properties explain the MEG sensor signal the best and furthermore,*when*they do so during the visual response.

In the present study, we modeled the predictions without the offset, that is, only the spatially specific responses (seeEq. 1). We did so with the reasoning that we baseline corrected our measured ERF data (setting their mean to zero), and hence should set the ‘mean’ of our pRF predictions (offset) to zero as well. However, we can see in the sensor ERF example (Fig. 4BandSupplementary Fig. 5for other sensor examples) that the mean across stimuli differs at later timepoints, indicating there are processes involved that could be captured by modeling the offset too. We therefore ran a separate analysis allowing the offset to be fitted in the same cross-validated manner, by adding the offset term to the scaled prediction inEq.1. In single sensors, sensor averages and across subjects (Supplementary Fig. 6A, B and C, respectively), we see that both models (with and without offset) perform equally well at early time windows, while the model*with*the offset leads to higher variance explained at later timepoints. Importantly, while the offset adds a parameter to the model, the fitting was cross-validated and the offset thus proves a valuable addition in explaining the signal at later timepoints.

Modulatory processes continuously influence the feedforward stream of information processing (V. A. Lamme et al., 1998) and by tweaking the model parameters of the forward modeling approach, we can test specific hypothesis about the dynamic mechanisms involved in visual processing. For example, by implementing other models than the simple 2D Gaussian model, we can investigate non-linear processes that influence visual processing, such as the center-surround organization, compressive spatial summation, and divisive normalization (Aqil et al., 2021;Kay et al., 2013;Zuiderbaan et al., 2012). We hypothesize that different models and with different parameter settings would more optimally explain signals at different latencies of visual processing. Specifically, several studies suggest that neurons’ receptive field properties are not fixed but change during the course of the visual response (Duan et al., 2019;Klein et al., 2014;Liu et al., 2022;Malone et al., 2007;Womelsdorf et al., 2008;Zirnsak et al., 2014). For example, as we employ a fixation task, attention may attract pRF positions towards the fixation (Klein et al., 2014). Likewise, coarse-to-fine theories hypothesize that pRF sizes reduce over time (Einevoll et al., 2011;Malone et al., 2007;Sasaki et al., 2010). Therefore, we did not use eccentricity or size to validate our analysis. How and when these pRF changes happen in the healthy human brain could be investigated with our approach, with the same temporal resolution as invasive electrophysiological studies.

Importantly, our approach could also be implemented in clinical neuroscience, to gain a better understanding of the potential changes in dynamics or timing deficits in various ophthalmologic and neurologic disorders. For example, in patients with schizophrenia, pRF center-surround configurations are altered (Anderson et al., 2017), affecting perception (Dakin et al., 2005) (seeDumoulin & Knapen (2018)for a review on changes in pRF properties in various clinical conditions). Our approach could reveal the timing of these altered spatial interactions in these clinical conditions.

A limitation of our method is that the modeling relies on an accurate head model, and different head models may yield different results (Huang et al., 1999;Lalancette et al., 2011). Similarly, the modeling assumes a stable head position during the recording, that is, that the relation and distance between head and sensor did not change throughout the recording. Inaccuracies in our head model estimation and instable head positions during recording might have led to underestimation of the variance explained by the pRF models. It is possible to correct for head movement, however, using Signal Space Separation (SSS;Taulu et al., 2005) and we speculate that this can further increase our model fitting accuracy.

An additional limitation is that participant must be measured twice, once using fMRI to estimate the pRF layout, and once with MEG to obtain the ERF responses. However, we expect that, at least for early visual regions, the pRF data may be replaced by pRF templates (Benson et al., 2012,^2014^).

Furthermore, not all MEG sensors were usable for fitting. Some sensors simply did not pick up signals from visual cortices because they were too far away from the occipital cortex (compare posterior vs. anterior located sensor inSupplementary Fig. 5). Other sensors, that did contain visual responses, could not distinguish between different pRF properties based on their predictions (Supplementary Fig. 7A). Consequently, they did not show a reduction in variance explained when shifting the pRF properties (Supplementary Fig. 7B). Lastly, in our present analysis, we used the pRF properties of*all*vertices in the visual cortex. The analysis can be extended to use the pRF parameters of a subset of pRFs, for example, single regions of interest such as V1, to evaluate the contributions of different visual areas.

In our perturbation analysis, we found that the variance explained for the shifted pRF polar angles did not reduce all the way to zero (Fig. 6). We propose that this is due to the fact that shifting a pRF will not move its dipolar pattern completely away from its most sensitive sensors and there will be remaining variance, especially visible when averaging over sensors (Fig. 6BandSupplementary Fig. 4). We observed that the reduction in variance explained differed over time, showing less reduction during later time windows, likely due to the fact that later time windows contain signal from pRFs with larger receptive fields.

In conclusion, we show that pRF models can predict event-related responses and we can examine pRF properties on the neuronal timescale. The forward pRF modeling approach offers a versatile method that will allow routine investigation of spatiotemporal dynamics of human pRFs, with both high spatial and temporal resolution.

## Supplementary Material

Supplementary Material

## Data Availability

Analysis code will be made publicly available on GitHub. Minimally preprocessed data will be made available upon request, due to The Netherlands and EU General Data Protection Regulation (GDPR) compliance.
