# Supplementary material for: Population receptive field models capture the event-related magnetoencephalography response with millisecond resolution
Source: Imaging Neurosci (Camb). 2024 Sep 18;2:imag-2-00285. doi: 10.1162/imag_a_00285 (PMC12290684; doi:10.1162/imag_a_00285)
Supplement: Supplementary Material [file imag_a_00285-supp.pdf]

Supplementary Material

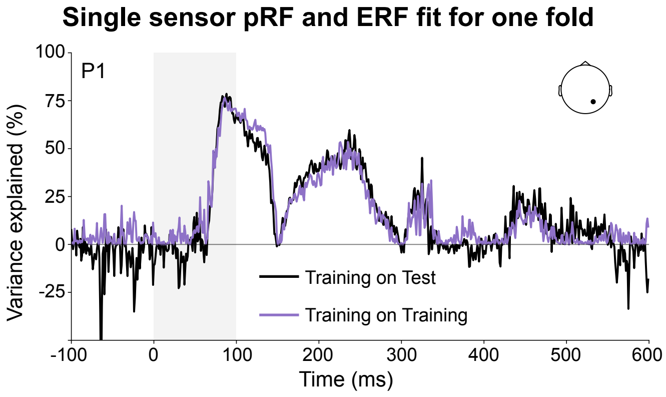

Supplementary Figure 1. Cross-validation can result in negative variance explained. Variance explained for an example sensor and example cross-validation fold in participant 1. For our analysis, we fitted the scaling factor (b1) on a 'training' ERF data set and computed the variance explained on a 'test' ERF data set (Eq. 2; black line). With this cross-validation procedure, variance explained can go negative, as it is bounded between -infinity and 1. Without cross-validation, i.e., the variance explained was computed on the 'training' ERF data set (purple line), the fit followed a similar trend but did not become negative, since non-cross-validated variance explained is bounded between 0 and 1.

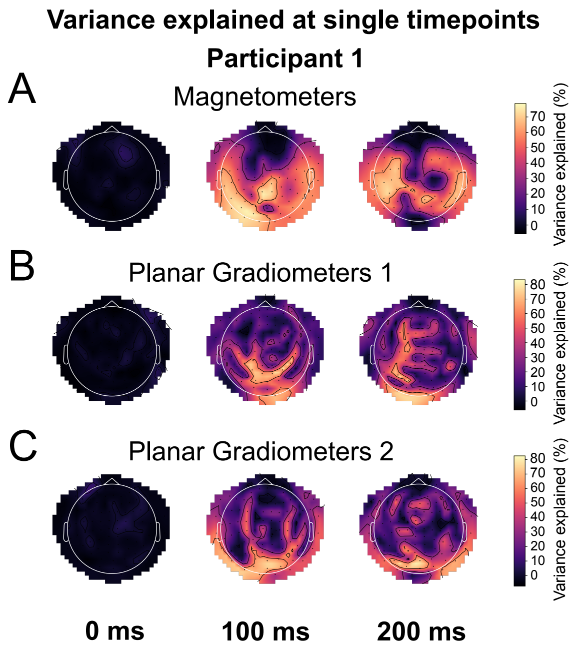

Supplementary Figure 2. Topographical distributions of variance explained for the three MEG sensor types. The pRF models explained the ERF signal across all three MEG sensor types: (A) magnetometers and (B, C) two orthogonal sets of planar gradiometers. We show three example timepoints (0, 100, and 200 ms after stimulus onset) for the same example participant 1 as shown in the Main Figures. The three sensor types have different sensitivity profiles in picking up the magnetic field. However, in our analysis, each sensor was treated independently and the distributions show that the pRFs explained the ERF signal across many sensors in all three sensor types.

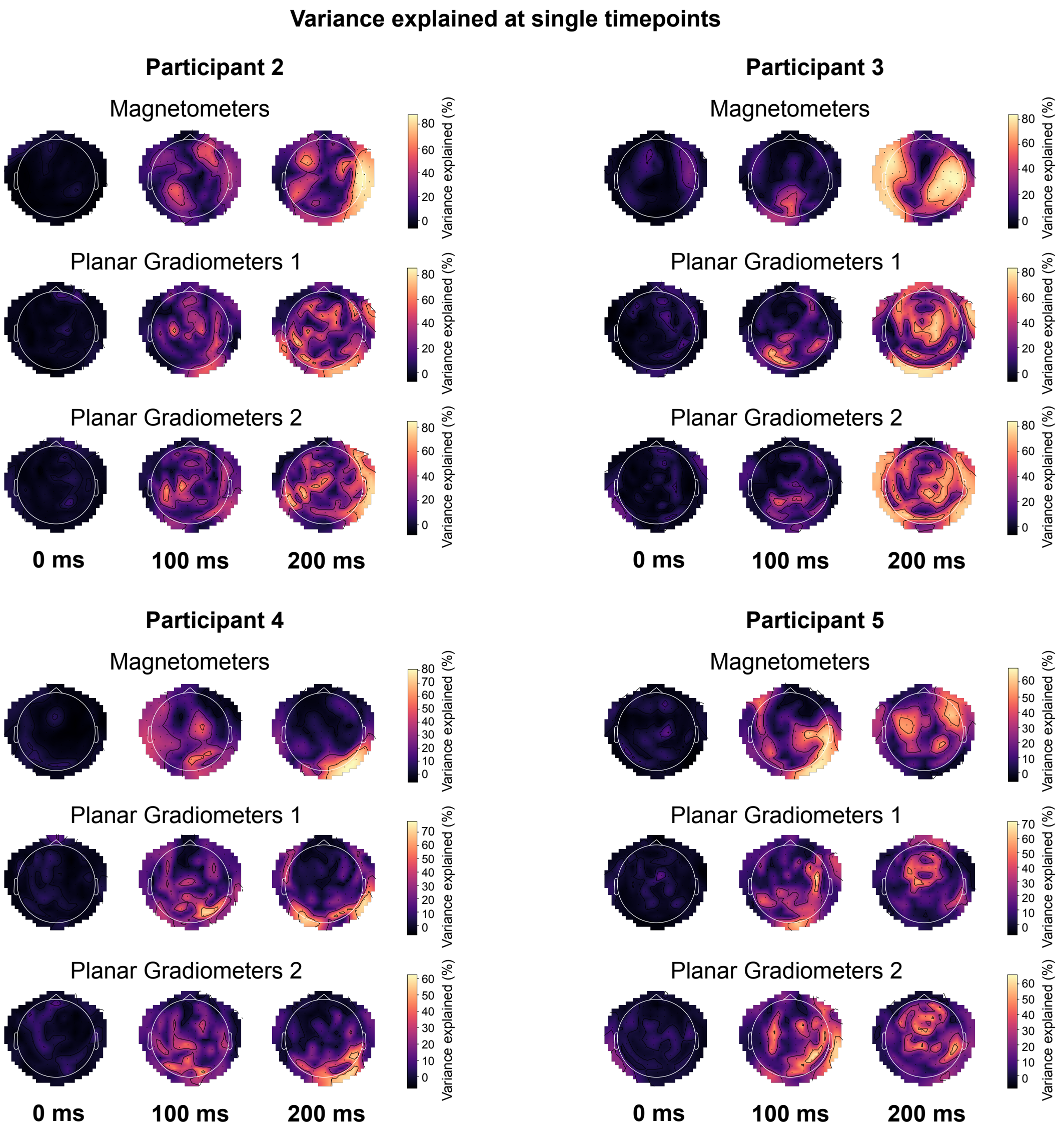

Supplementary Figure 3. Topographical distributions of variance explained for the three MEG sensor types for the other participants. The pRF models explained the ERF signals at different timepoints (three example timepoints 0, 100 and 200ms after stimulus onset shown) across participants in all three MEG sensor types. The exact spatial distribution of sensors that were captured by the pRF models differed across subject due to the different head anatomies and respective positions in the MEG helmet during the recording.

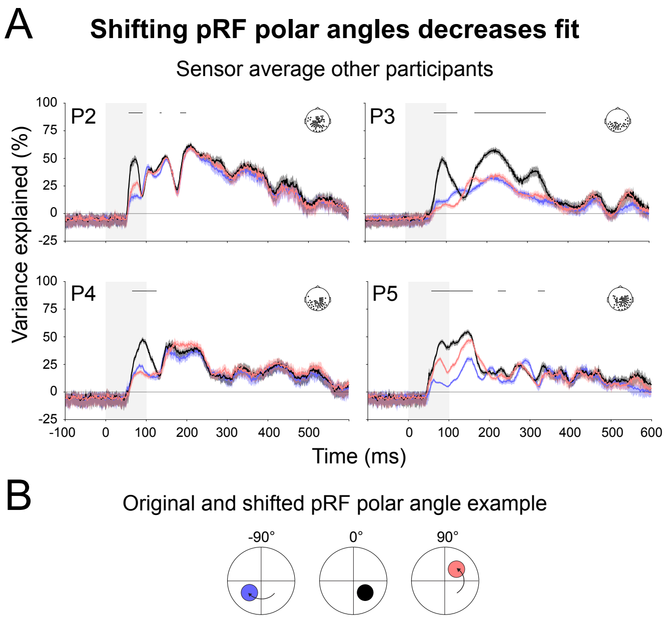

Supplementary Figure 4. ERF sensitivity to polar angles across other participants. (A) Average fit of ERF and original pRF predictions (black line, middle circle in B) and pRFs shifted 90 degrees clockwise (blue line, left circle in B) and counterclockwise (pink line, right circle in B), for participants 2 to 5 in 'earliest' sensors. Solid lines and fills indicate the medians and 95% confidence intervals over cross-validation folds. The variance explained decreased when the pRF polar angles were shifted away from the fMRI-estimated angles. Horizontal black lines on top mark the latencies where the fits were significantly decreased, i.e., the CI of the shifted pRF fits were not overlapping with the CI of the original fit. We observed a reduction of variance explained for every participant, particularly for the window 75-150ms, but also for the later time window in participants 3 and 5. (B) Illustration of pRF polar angle shifts.

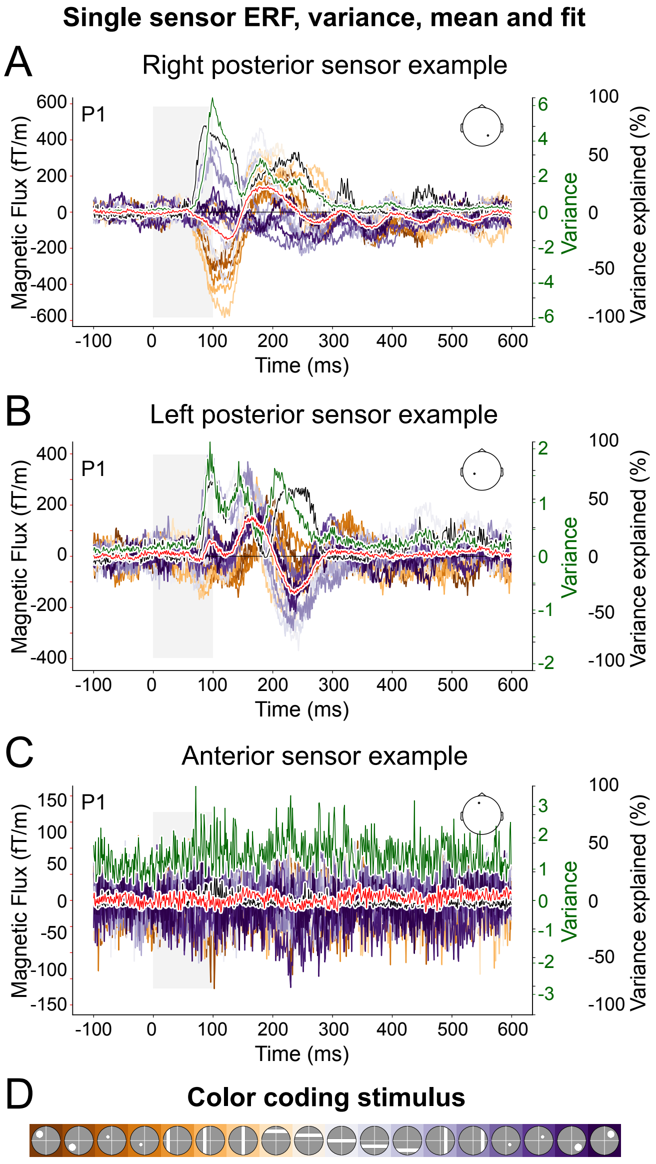

Supplementary Figure 5. ERF, ERF-variance, ERF-mean and pRF-ERF fit for sensor examples. All figures show ERFs color-coded according to visual space from left (orange) to right (purple) (see (D) for stimulus color-codes), with an overlay of variance explained (black) for the given sensor example, the variance of the ERF values at a given timepoint (green), and the ERF's mean (red). The sensor locations are marked by the dot on the head layout in the upper right corner. We show example sensors in participant 1 (note the different y-scales for the different sensors). (A) Posterior example sensor located over the right occipital cortex of the participant. (B) Posterior example sensor located over the left occipital cortex. (C) Anterior sensor example located over the participant's frontal cortex.

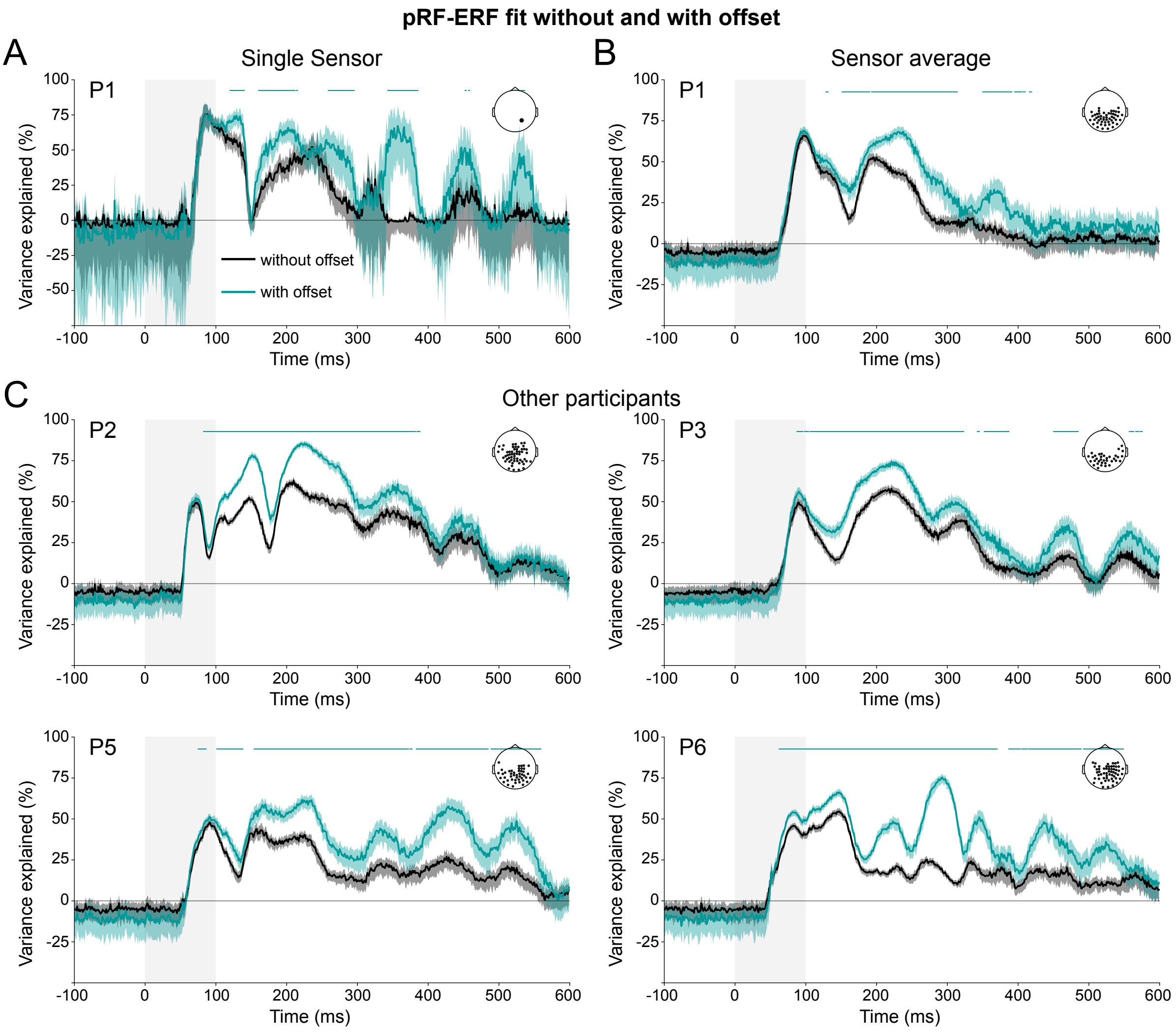

Supplementary Figure 6. **pRF-ERF fit without and with offset (b0).** For our main analyses, we set the offset to zero, since the ERFs were baseline corrected, resulting in a mean around zero. Here, we repeated the analyses where the offset was allowed to be modelled, that is, we added the fitted b0 term to the sensor predictions when calculating the variance explained of the test set. (A) Variance explained time course of one example sensor when the variance explained was calculated with the offset (turquoise line) as compared to without (black line). (B) Sensor average over the 'earliest sensors' for the same participant, and (C) Sensor average 'earliest sensors' for the other four participants. While fitting without or with offset resulted in similar variance explained values for the first peak, the addition of the offset increased the variance explained to varying degrees at later timepoints.

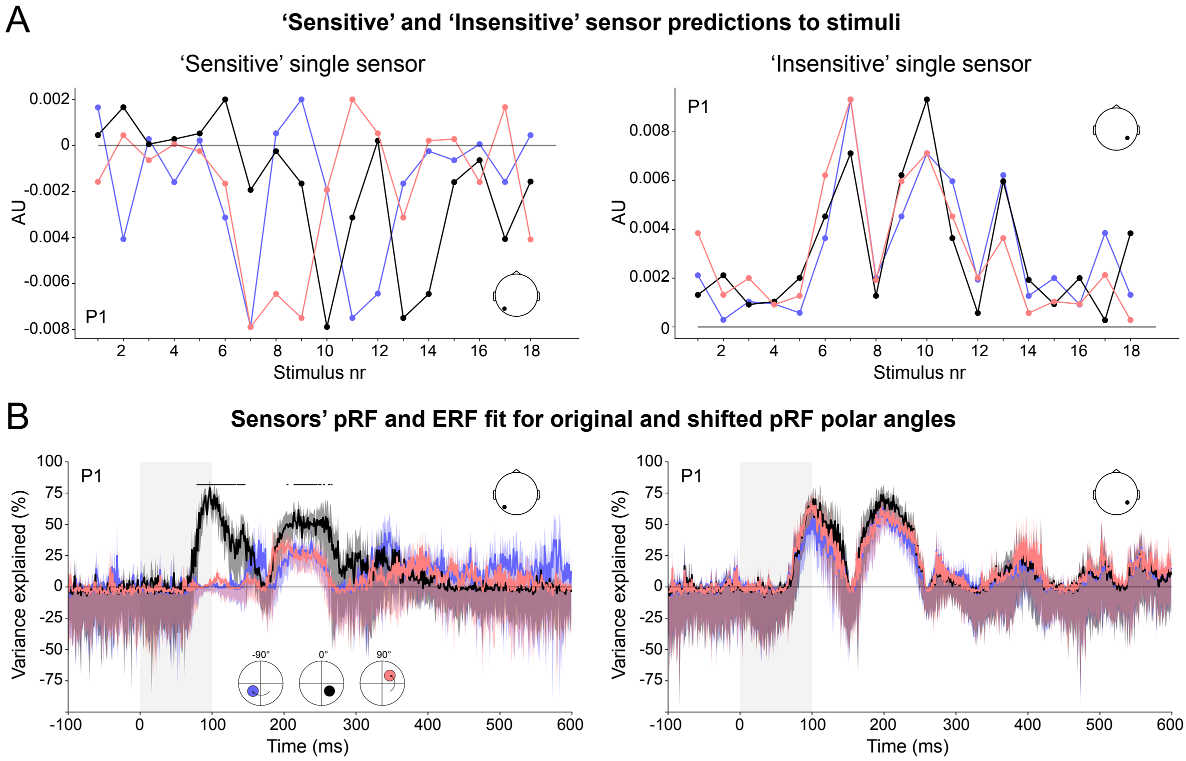

Supplementary Figure 7. Sensitivity to pRF polar angle changes. (A) Sensor predictions to the eighteen MEG stimuli (x-axis) based on the original pRF polar angles (black line) and their shifted versions (blue and pink for -90 and 90 degrees shifts, respectively) for an example sensor in participant 1 that was 'sensitive' to pRF polar angle changes (left panel) and an example sensor that was not sensitive (right panel). (B) Variance explained for the original pRF polar angles and their shifted versions for the same 'sensitive' and 'insensitive' sensor. Solid lines and fills indicate the medians and 95% confidence intervals over cross-validation folds. Black horizontal lines mark the timepoints at which the shifted polar angle fits were significantly decreased as compared to the original pRF fit (i.e., non-overlapping confidence interval). We found a significant reduction in variance explained for the shifted pRF polar angles for the 'sensitive' sensor, but not for the 'insensitive' sensor.
